# Components of Guilt and Psychopathological Features in Eating Disorders: Preliminary Results

**DOI:** 10.1192/j.eurpsy.2026.12027

**Published:** 2026-07-31

**Authors:** F. Raffone, D. Atripaldi, M. Carfagno, E. Barone, A. Capasso, V. Martiadis

**Affiliations:** 1 Department of Mental Health, Asl Napoli 1 Centro; 2 Department of Advanced Medical and Surgical Sciences; 3 Department of Psychiatry, University of Campania “L. Vanvitelli”, Naples, Italy

## Abstract

**Introduction:**

Guilt is a complex emotion frequently implicated in the psychopathology of eating disorders (EDs), yet its specific dimensions have rarely been analyzed in relation to clinical and diagnostic profiles.

**Objectives:**

This study aimed to explore the associations between the dimensions of guilt, measured with the Multidimensional Orientation Guilt Scale (MOGS), and clinical and psychological features assessed with the Eating Disorder Inventory-2 (EDI-2) in a sample of patients with EDs.

**Methods:**

Sixty-six patients diagnosed with Anorexia Nervosa, Bulimia Nervosa, or Binge Eating Disorder were included. All participants completed the MOGS and the EDI-2. Associations between variables were analyzed using Spearman correlations. A stepwise multiple linear regression was conducted to identify predictors of EDI-2 psychopathological dimensions.

**Results:**

Significant correlations between guilt subscales and diagnosis included MNV (ρ=0.47, p<0.01), MODI (ρ=0.30, p<0.01), EMPATHY (ρ=0.32, p<0.01), and HARM (ρ=0.32, p<0.01). Several EDI-2 subscales were significantly associated with guilt dimensions, particularly EDI_B with MNV (ρ=0.25, p=0.02), EMPATHY (ρ=0.23, p=0.03), deontological guilt (ρ=0.23, p=0.03), and altruistic guilt (ρ=0.21, p=0.04). In the regression model, the “Asceticism” subscale of the EDI-2 was significantly predicted by the EMPATHY dimension of the MOGS (β=0.31, p=0.01), with an overall significant model (R²=0.10, p=0.01).

**Conclusions:**

These preliminary results suggest that different dimensions of guilt are associated with specific psychopathological profiles in EDs, with particular relevance for the empathic dimension. Structured assessment of guilt may have clinical utility in both the diagnostic and therapeutic processes for patients with eating disorders.

**Disclosure of Interest:**

None Declared

